# *neuroConstruct*: A Tool for Modeling Networks of Neurons in 3D Space

**DOI:** 10.1016/j.neuron.2007.03.025

**Published:** 2007-04-19

**Authors:** Padraig Gleeson, Volker Steuber, R. Angus Silver

**Affiliations:** 1 Department of Physiology, University College London, Gower Street, London WC1E 6BT, United Kingdom

**Keywords:** SYSNEURO

## Abstract

Conductance-based neuronal network models can help us understand how synaptic and cellular mechanisms underlie brain function. However, these complex models are difficult to develop and are inaccessible to most neuroscientists. Moreover, even the most biologically realistic network models disregard many 3D anatomical features of the brain. Here, we describe a new software application, *neuroConstruct*, that facilitates the creation, visualization, and analysis of networks of multicompartmental neurons in 3D space. A graphical user interface allows model generation and modification without programming. Models within *neuroConstruct* are based on new simulator-independent NeuroML standards, allowing automatic generation of code for NEURON or GENESIS simulators. *neuroConstruct* was tested by reproducing published models and its simulator independence verified by comparing the same model on two simulators. We show how more anatomically realistic network models can be created and their properties compared with experimental measurements by extending a published 1D cerebellar granule cell layer model to 3D.

## Introduction

The characteristic 3D structures of brain regions like the cerebellum, hippocampus, and cortex and the complex connectivity between and within them are thought to play a key role in determining how information is distributed and processed in the brain. Moreover, neuronal classes exhibit unique morphologies, and modeling studies have shown that the shape of the dendritic tree affects the electrical behavior (Mainen and Sejnowski, 1996; van Ooyen et al., 2002; Vetter et al., 2001) and that the spatial pattern of synaptic contacts influences how signals are integrated (Blomfield, 1974; Destexhe and Pare, 1999; Jarsky et al., 2005; Markram et al., 1997; Mel, 1993; Rall et al., 1967). Neuronal signaling is not only restricted to point-to-point synaptic transmission but is also mediated by diffuse messengers including classical neuromodulators (e.g., ACh, 5-HT), nitric oxide (Jacoby et al., 2001), cannabinoids (Alger, 2002; Wilson and Nicoll, 2002), and neurotransmitters (Mitchell and Silver, 2000). The signal processing carried out by an individual neuron is therefore determined by both its morphology and the 3D structure of the network in which it is embedded.

Understanding how complex brain structures and the myriad of underlying mechanisms interact to produce higher-level functions will require the help of network models with biologically realistic features. Models that use compartmental neurons, Hodgkin-Huxley type membrane conductances, and semirealistic synaptic connectivity have been used to explore the potential mechanisms underlying synchronous activity (Davison et al., 2003; Maex and De Schutter, 1998), cortical oscillations (Traub et al., 2005), hippocampal memory (Kunec et al., 2005), and temporal coding (Buonomano, 2000). They have also provided insights into potential causes of epileptiform activity in dentate gyrus (Santhakumar et al., 2005) and cortex (Bush et al., 1999; Traub et al., 2005). However, virtually all such models utilize simplified synaptic connectivity, featuring abstract neurons connected in either one dimension (Maex and De Schutter, 1998; Santhakumar et al., 2005) or two-dimensional layered structures (Medina and Mauk, 2000; Schweighofer and Ferriol, 2000).

The development of more biologically realistic network models that include explicit 3D information would allow direct comparison of the model structure with anatomical measurements. Such network models would also allow direct comparison of the spatiotemporal properties of simulated neural activity with experimental measurements using multielectrode recordings (Buzsaki, 2004; Nicolelis and Ribeiro, 2002) or two-photon imaging of activity in blocks of tissue (Ohki et al., 2005; Stosiek et al., 2003). Moreover, they could be extended to simulate volume signaling and brain metabolism. While the development of such 3D network models is theoretically possible with current simulators such as NEURON (Hines and Carnevale, 1997) and GENESIS (Bower and Beeman, 1997), and some preliminary attempts have been made (Berends et al., 2004; Howell et al., 2000), considerable technical difficulties remain. These include a requirement for algorithms that can create the highly nonuniform 3D synaptic connectivity observed in biological networks (Song et al., 2005; Sporns and Kotter, 2004; Yoshimura and Callaway, 2005), a method for verifying connectivity and routines for analyzing network behavior. Indeed, the absence of such tools has prevented the development and use of more biologically realistic 3D network models.

A more integrated understanding of brain function will require closer interaction between experimental and theoretical neuroscientists (De Schutter et al., 2005; Destexhe and Marder, 2004; Segev and London, 2000). At present, communication between these groups, and even between individual theoreticians, is hampered by poor accessibility and interoperability of models. Although single-cell and network models are available on public databases (Hines et al., 2004), their utility as research tools is often restricted to those familiar with the specialist scripting languages, which are simulator specific. For example, a neuronal model written in NEURON script cannot be used as part of a GENESIS simulation, thereby limiting its interchange and reuse. Although graphical user interfaces (GUIs) have improved the accessibility of single-cell models, networks remain inaccessible to most neuroscientists.

We have developed a new software application, *neuroConstruct*, that facilitates the creation and analysis of networks of multicompartmental neurons in 3D space. Automated cell placement and generation of synaptic connectivity, together with 3D visualization, allow the creation and verification of models with greater anatomical realism than achieved previously with script files. A GUI and the automated generation of code for NEURON or GENESIS allow network models to be built, modified, and run without programming, enhancing their accessibility. Model reuse and interchangeability are facilitated through the implementation of a simulator-independent model description based on NeuroML standards (Crook et al., 2007; Goddard et al., 2001). We describe and test the functionality of *neuroConstruct* and extend a 1D model of the granule cell layer of the cerebellar cortex (Maex and De Schutter, 1998) to 3D, thereby providing an example of behavior, previously observed in vivo (Vos et al., 1999), that could not be captured in the original 1D model.

## Results

### Outline of Application

*neuroConstruct* is a JAVA-based software tool for constructing neural network models with many biologically realistic features. These include realistic cell morphologies, voltage- and ligand-gated ion channels, cell densities, synaptic connectivity patterns, and gross 3D structures of different brain regions. Cell and network models can be built through the *neuroConstruct* GUI and automatically simulated on either the NEURON or GENESIS platform. The latest version of *neuroConstruct*, including the models described in this paper and a number of tutorials, is freely available for download from http://www.neuroConstruct.org.

**Figure 1:**
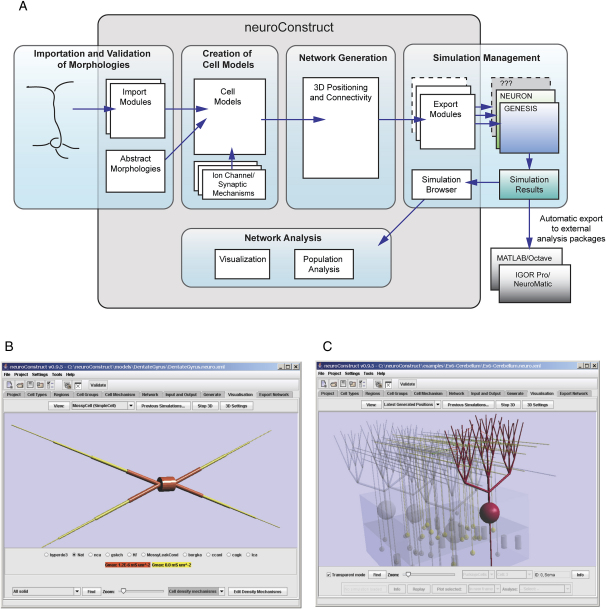
Overview of *neuroConstruct* (A) Schematic view of the main functionality of *neuroConstruct*. (B) Main *neuroConstruct* GUI showing a single abstract cell with a Na^+^ channel conductance density that varies on different parts of the cell membrane. (C) Main interface to *neuroConstruct* showing the visualization of a simple network using the transparency feature to highlight a single cell and its connections.

*neuroConstruct's* functionality can be grouped into five main areas (Figure 1A).

#### (1) Importation and Validation of Morphologies

Reconstructed neuronal morphologies, commonly used in conductance-based neuronal models, can be imported into *neuroConstruct* in various formats (e.g., Neurolucida) and automatically checked for errors. More abstract morphologies with a smaller number of compartments can also be created manually (Figure 1B).

#### (2) Creation of Simulator-Independent Conductance-Based Cell Models

Modeling of detailed cellular mechanisms, such as the conductance changes produced by voltage- and ligand-gated ion channels, is essential for reproducing the complex behavior of real neurons. Cell mechanisms can be defined in *neuroConstruct* in a simulator-independent format and cell models created by specifying the complement and density of these on the cell membrane (Figure 1B).

#### (3) Network Generation

Once cell models have been created in *neuroConstruct*, they can be placed within a region of 3D space at a specified density (Figure 1C). Layered structures, such as the cortex, can be created from stacks of contiguous regions. Once the cells are arranged, synaptic connections can be generated according to specified sets of rules.

#### (4) Simulation Management

Network simulations are carried out by automatically generating script files for the simulator packages NEURON or GENESIS and the results stored in text files.

#### (5) Network Analysis

Simulations can be loaded back into *neuroConstruct* for visualization and analysis. For more specialized analyses, script files are created that allow data to be imported into two common numerical analysis packages.

### Description of Functionality and Validation of Application

#### Neuronal Morphology

**Figure 2:**
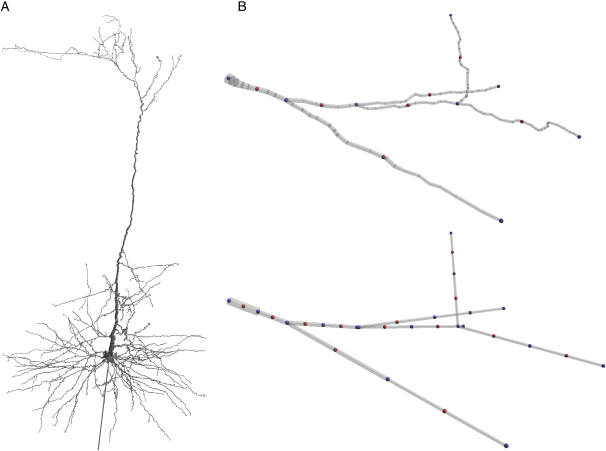
Detailed Cell Morphologies in *neuroConstruct* (A) A detailed reconstruction of a neocortical pyramidal cell (Mainen et al., 1995) imported into *neuroConstruct* from a NEURON morphology file. (B) Detail of a small part of a dendritic tree. Upper view: all 3D detail present in the original morphology file. Sections (between the blue spheres) contain a number of 3D points with associated diameter, each of which is the endpoint of a segment (small gray conical frusta). NEURON uses this information to compute membrane area and axial resistance, but only performs numerical integration at specific locations (red spheres; determined by *nseg*). Lower view: simpler representation of cell structure with fewer segments for mapping to GENESIS (Experimental Procedures).

Neuronal models with complex morphologies have been used to investigate various aspects of synaptic integration and neuronal excitability (De Schutter and Bower, 1994; Destexhe and Pare, 1999; Hanson et al., 2004; Jarsky et al., 2005; Mainen et al., 1995; Migliore et al., 1995; Poirazi et al., 2003; Vetter et al., 2001), and public databases have been produced that contain examples of anatomical reconstructions of stained neurons (Ascoli, 2006; Cannon et al., 1998). However, using such morphology files in compartmental models is complicated by the fact that they are often in different formats, their anatomical and electrical compartments are not equivalent and there are subtle differences in how the morphological information is used by different simulators. To overcome these problems, *neuroConstruct* can import and visualize morphology files with different formats (Figure 2A), including Neurolucida (^∗^.asc; http://www.mbfbioscience.com/neurolucida), GENESIS readcell compatible format (^∗^.p), most NEURON/ntscable generated morphology files (^∗^.nrn or ^∗^.hoc), and Cvapp (^∗^.swc) format (Cannon et al., 1998). The simulator-independent representation of the morphology used in *neuroConstruct* allows the same model to be mapped onto different simulator structures (Experimental Procedures) and is closely related to MorphML (Crook et al., 2007), a new standard for describing neuronal morphologies. MorphML is based on XML (extensible markup language), and is the core of level 1 of the NeuroML framework (Crook et al., 2007; Goddard et al., 2001; http://www.neuroml.org). *neuroConstruct* also has a recompartmentalization function that can reduce the total number of compartments while conserving morphological features such as total membrane area and section length (Figure 2B; Experimental Procedures), thereby speeding up simulations (see Figure S1 in the Supplemental Data available with this article online). Large-scale networks of thousands of neurons often use simplified cell models with fewer compartments to minimize the computational overhead (Santhakumar et al., 2005; Traub et al., 2005). These can be created manually in *neuroConstruct* and are handled in the same way as more detailed cells.

#### Cell Mechanisms

Neuronal signaling is mediated by a variety of subcellular, membrane, and synaptic mechanisms. Models of cellular mechanisms can be simple, such as a synaptic conductance waveform, or more complex, like Hodgkin-Huxley type formulations of voltage-gated conductances, which depend on both voltage and time, and their conductance density can be nonuniformly distributed over the cell membrane. Such models form a core part of any conductance based neuronal simulation, but their implementation is one of the more complicated aspects of using existing simulation packages. Although the mathematical framework used to describe such mechanisms (e.g., maximum channel conductance, reversal potential, rate equations) is general and familiar to many neuroscientists, implementation of these in NEURON or GENESIS involves use of a platform-specific programming language.

**Figure 3:**
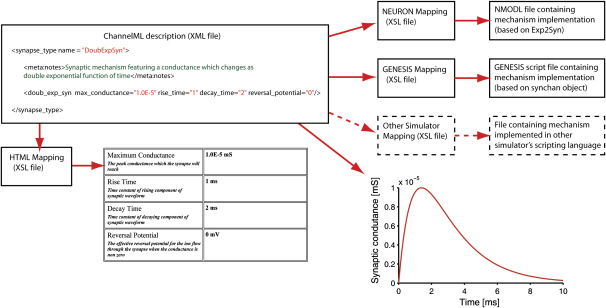
Use of ChannelML for Specifying Cellular Mechanisms A ChannelML file (the code fragment shows the parameters needed to specify a double exponential time course synapse) can be converted into script files in the native language of various neuronal simulators (currently NEURON and GENESIS), using an XSL file for each mapping. HTML representations of the XML file provide a more readable view of the mechanism and associated metadata. Plots can be generated to view the mechanism's properties.

Models of cell mechanisms are implemented in *neuroConstruct* using a ChannelML-based description, which forms part of level 2 of the NeuroML framework (Crook et al., 2007). Figure 3 shows a ChannelML file describing a synaptic conductance mechanism and how it can be used. It consists of an XML file containing the physiological parameters in a structured format that can be validated against a specification, reducing the probability of errors. Information in XML files can easily be transformed into other formats with an XSL (extensible stylesheet language) mapping file (Figure 3). We have created XSL files which map ChannelML descriptions of cell mechanisms onto NMODL (Hines and Carnevale, 2000) format for NEURON and onto the appropriate object in a GENESIS script file. The simulator-independent XML format promotes compatibility with other simulators: for each newly supported simulator, a single XSL file needs to be created which maps the files onto its specialized format. The nature of XML also allows translation of the file into HTML, allowing the cell mechanism to be presented in an easy-to-read format, facilitating online archiving.

A number of ChannelML templates are included with *neuroConstruct*. Parameters in these models can be easily modified through the GUI to match the channel kinetics in a particular cell type, either from a published model or directly from experimental measurements of these parameters (e.g., m_∞_, τ_m_, h_∞_ and τ_h_ for a Hodgkin-Huxley type model of a Na^+^ channel). However, ChannelML specifications are still in development, and some cell mechanism types are not yet covered. To allow for unsupported models and to provide greater backward compatibility, files in NMODL (^∗^.mod files) or GENESIS script (e.g., tabchannel based) can be incorporated into cell models created with *neuroConstruct*, but this makes the model simulator specific. For example, synaptic plasticity mechanisms and Markov models can be incorporated into a *neuroConstruct*-based model by inserting an NMODL file for simulation in NEURON.

The use of different systems of units can lead to errors in translation between the different simulators. GENESIS uses a consistent set of either SI units or physiological units (ms, mV, etc.), whereas NEURON has its own system based on physiological units. Conversion between these systems of units is handled automatically by *neuroConstruct*.

#### Creation of Cell Models

Once a cellular morphology has been imported or created in *neuroConstruct*, groups of sections can be defined to distinguish axons, somata, and dendrites. Subgroups of sections such as proximal, oblique, and apical dendrites can also be defined. The distribution of cellular mechanisms can then be specified for each cell region. For example, a nonuniform channel density can be implemented by varying the conductance density in each group (Figure 1B). Ion-concentration mechanisms (e.g., activity-dependent intracellular Ca^2+^ concentrations) can also be added in this way, as can passive electrical properties (specific membrane capacitance and specific membrane/axial resistance), allowing spine densities to be simulated without additional compartments. New cell models can be created from experimental or published data using *neuroConstruct* by importing/creating cell morphologies and modifying existing ChannelML templates or adding native code. However, the painstaking process of making a detailed multicompartmental cell model from scratch often involves automated optimization of parameters and access to all model variables, which requires coding with a command-line-based simulator or another program (e.g., MATLAB or Python; see Discussion).

#### Comparison of a Cell Model on Two Independently Developed Simulators

**Figure 4:**
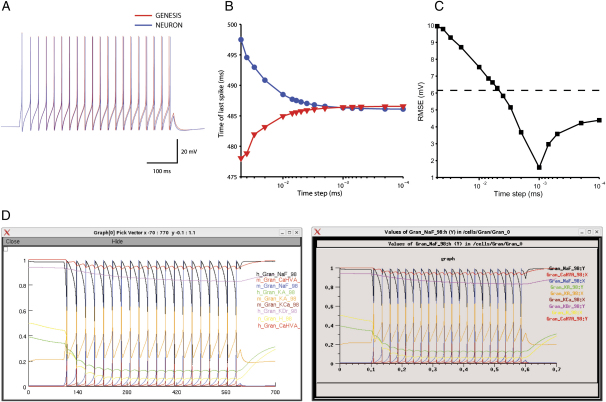
Test of the Simulator Independence of a *neuroConstruct* Model (A) Membrane potential responses to a 500 ms current pulse of 10 pA at a simulation time step of 0.01 ms for a granule cell model (Maex and De Schutter, 1998) implemented in *neuroConstruct* using ChannelML and run on NEURON and GENESIS. (B) Dependence of timing of last action potential on integration time step. (C) Dependence of the root-mean-square (rms) of the difference between traces on integration time step. The minimum at 0.001 ms is due to the peaks overlapping before converging at slightly different times in each simulator. The dotted line shows the rms error between the GENESIS model and one with a 1% difference in Na^+^ conductance density. (D) Values of some of the internal state variables as a function of time (ms) displayed as screenshots from NEURON (left, time units ms) and GENESIS (right, s).

To test *neuroConstruct*'s simulator-independent representation of channel mechanisms, we have recreated a published model of a cerebellar granule cell (GrC), originally written in GENESIS (Maex and De Schutter, 1998), and compared its properties on NEURON and GENESIS. The model contains multiple ion conductances, including a fast inactivating Na^+^ conductance, three K^+^ conductances (delayed rectifier, A type, and Ca^2+^ dependent), a high-voltage activated Ca^2+^ channel, and a hyperpolarization activated H conductance. It also has a passive leak conductance and an exponentially decaying pool of calcium. In the *neuroConstruct* version, all channels are specified in ChannelML. Figure 4A compares membrane potential during a depolarizing current step for simulations run on GENESIS and NEURON. The timing of the final action potential (AP) in the train depended strongly on the integration time step, and the two simulators converged to values separated by less than 1 ms after a 500 ms simulation run (Figure 4B). The root mean squared (RMS) difference between the voltage traces decreased over the range of commonly used simulation time steps (Figure 4C), indicating the traces as a whole, not just the final spike, converged. The internal state variables were also similar on each of the simulators under these conditions (Figure 4D). To investigate how significant the difference between the simulators was, we compared the RMS error to that when the Na^+^ conductance had been altered by only 1% (Figure 4C, dashed line). The error due to the difference in simulator choice is smaller than this error, and thus insignificant when taking into account biological variability and the uncertainty in the conductance density.

We also compared simulations on NEURON and GENESIS of a morphologically complex neuron (Mainen et al., 1995; Figure 2A), to test the simulator-independent representation of both the channel mechanisms and the morphology. Simulations on both simulators closely reproduced the results of the original model (Figure S1). These results demonstrate that model descriptions in *neuroConstruct* are simulator independent.

#### Cell Placement in 3D

The gross anatomy of a brain region is generated in *neuroConstruct* by defining 3D regions in which specific cell types are placed. Regions can currently be boxes, spheres, cylinders, or cones, and multiple regions can be used to create composite structures such as the layers found in the cerebellum (Figure 1C) and cortex. Cells in *neuroConstruct* are arranged in cell groups, which are created by specifying the cell type, the 3D region in which the cells are found, and the packing pattern used to fill the space. Possible packing patterns include the following: cubic close packing for maximum density in 3D space, evenly spaced packing in 3D with cell body centers aligned, hexagonal planar patterns, single cells placed at a specified location, and cells placed in a one-dimensional line. However, for many brain regions, random cell placement is more realistic (Figure 1C). The number of cells in a specified region can be set, and whether cells should avoid the space occupied by existing cell bodies or can overlap can be specified. This allows cell densities to be matched to experimentally measured values for a particular brain region.

#### Generation of Synaptic Connectivity Patterns

**Figure 5:**
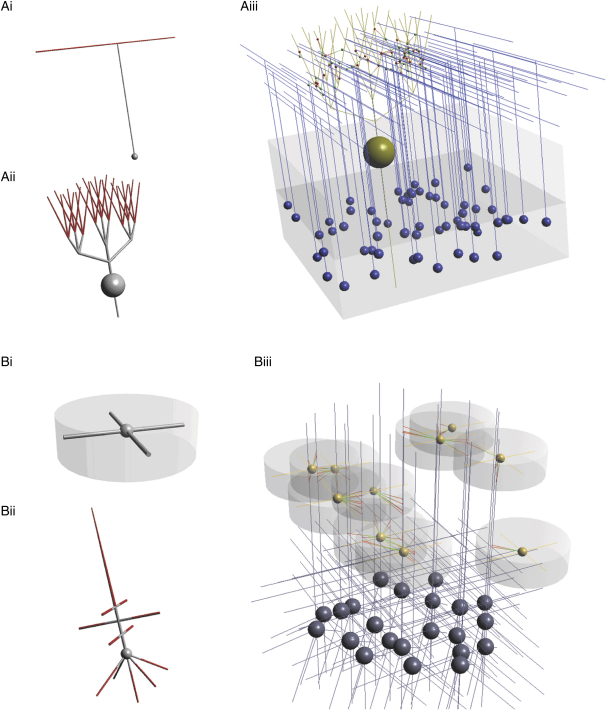
Connectivity Schemes Used to Generate Network Connections between Cell Groups in *neuroConstruct* (A) Simplified morphology of a GrC (i) including soma and axon. Parallel fiber sections, highlighted in red, indicate presynaptic sections where synapses are permitted. Simplified morphology of a PC (ii) with red dendritic sections showing postsynaptic sections where synapses are permitted. Connections between multiple GrCs and a PC made using the morphology-based connection algorithm (iii). Green and red spheres show the sites of pre- and postsynaptic connection, respectively. (B) Simplified morphology of a cortical interneuron (i) including soma, dendrites, and a cylindrical volume (gray shading) defining boundaries of the axonal arbor. Simplified morphology of a cortical pyramidal cell (ii) with red dendritic sections showing postsynaptic sections where synapses are permitted. Three-dimensional connections between multiple interneurons and pyramidal cells made using the volume-based connection algorithm (iii). Sites of pre- and postsynaptic connections are linked by lines changing from green to red.

Once cells are arranged in 3D, synaptic connections can be created between cell groups or within a single cell group. The set of rules specifying synaptic connections between cell groups and the associated type of synaptic mechanism is termed a network connection. There are two different ways in which network connections can be generated in *neuroConstruct*. The first, morphology-based connections, works by defining regions on pre- and postsynaptic cells where synaptic connections are allowed. Several other parameters can also be set, including the number of synapses per cell and the maximum and minimum connection lengths (Experimental Procedures). Figure 5A shows how the morphology-based connection algorithm can be used to generate connections between simplified models of GrCs and Purkinje cells (PCs) in the cerebellar cortex. GrC axons consist of an ascending axon and a T-shape bifurcation giving rise to a so-called parallel fiber (PF), which passes through the planar dendritic arbor of PCs (Figures 5Ai and 5Aii). The PF sections were specified as potential locations of presynaptic connections, and a subset of the PC dendritic sections (Figures 5Ai and 5Aii, red) were specified as possible postsynaptic connection locations. In this case, the number of connections between pre- and postsynaptic neurons was constrained to a maximum of one (Figure 5Aiii).

The second algorithm, which we term a volume-based connection, is designed for cases where the axon is a dense, highly arborized structure, distributed over a specific region of space, as commonly found in the cortex. Figure 5Bi shows a simplified model of a cortical interneuron and a cylindrical volume that defines the bounds of its axonal arborization. The diagram of a simplified pyramidal cell in Figure 5Bii shows the subset of its dendritic tree where connections of that type are permitted. When the cells are placed in 3D, regions of the dendritic trees of a number of pyramidal cells which fall within the axonal arbor of the interneurons are potential candidates for connections. These are made randomly based on the user-defined connectivity conditions, which include the number of connections per source cell and the maximum number of connections on each target cell. Other shapes including cones and spheres can be used to define the 3D bounds of axonal arborizations. The probability of making a synaptic connection within this volume can also be nonuniform (Experimental Procedures), allowing a preference for local connections.

There is also provision to introduce randomness into the amplitudes of the synaptic conductances and their onset delays for both connectivity algorithms. Moreover, the spatial location of the network elements can be used to simulate interesting 3D phenomena. We have used the ability to insert native code in *neuroConstruct* and the 3D spatial information contained within models to develop a very simple model of extracellular diffusion. Figure S2 shows a 3D network model with a diffusible signaling molecule that transiently inhibits the AMPA synaptic conductances. Changes in synaptic weight can be visualized by replaying the simulation in *neuroConstruct* or exported and plotted as a function of distance from the source. Although preliminary, this simulation illustrates the potential for creating models of volume-based signaling involving NO, cannabinoids, or neurotransmitters together with the supply and removal of metabolic factors.

#### Network Visualization and Input Properties

As network models can vary widely in size, there are a number of functions in *neuroConstruct* to facilitate the clear display of large networks, cells with complex morphologies, and individual synaptic connections. These include showing the dendrite and axon as lines (Figures 5Biii) or just showing ball-shaped somata (Figure 8A), rather than the full 3D structure of each cell (Figure 2). An adjustable transparency mode is available for visualizing cells deep within large networks. This allows an individual cell, defined groups of cells, or cells within a defined region to be highlighted (Figures 1C and 7B). These functions allow cells to be viewed in networks of thousands of cells on most standard desktop computers. Functions for analyzing the anatomical properties are also available within *neuroConstruct* (see Extension of the 1D Cerebellar Granule Cell Layer Model to 3D). The cell placement and network connectivity can be imported and exported in NetworkML format (level 3 of the NeuroML framework), allowing networks created with other applications to be loaded into *neuroConstruct* for visualization and use in simulations.

The external activation of a network with defined patterns of stimuli can be achieved in several ways. Cell groups can receive two main types of inputs: current steps of specific duration, delay, and amplitude or random trains of synaptic inputs, with a defined input frequency or a range of frequencies. Both of these types of input can be applied to all cells in a group, to a fixed percentage of cells, or to cells inside or outside a defined 3D region. The last option can be used to apply spatially organized input patterns to networks.

#### Verification of Network Models Implemented in *neuroConstruct*

**Figure 6:**
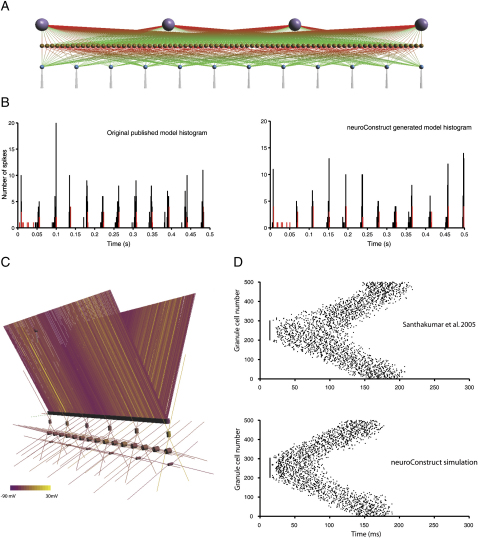
Implementation of Existing Network Models in *neuroConstruct* (A) Visualization of a 1D GrC layer network model from Maex and De Schutter (1998). MFs (bottom) are connected via excitatory synapses to GrCs (middle), which are in turn connected to GoCs (top). The GrCs receive inhibitory connections from GoCs. (B) Spike time histograms (bin size, 1 ms) as produced by the script files released with the original publication (left) and for the *neuroConstruct* model of the network (right). Spikes for the GrCs are in black and the GoCs are in red. (C) Replication of a network model of the dentate gyrus (Santhakumar et al., 2005). The model consists of (from the top down) 500 GrCs with two dendritic branches, 6 basket cells, 15 mossy cells, and 6 hilar cells. The 10,000+ synaptic connections have been removed for clarity. The network receives a brief perforant path focal stimulation, mainly on the central 100 GrCs. Cell coloring reflects network activity 110 ms after stimulation. (D) Raster plots of dentate gyrus GrC activity in the original published model and in the *neuroConstruct* implementation of the network.

We verified the ability of *neuroConstruct* to generate accurate network models by implementing two published network models and comparing the behavior of the *neuroConstruct* versions to the original models. We first tested the conductance-based model of the cerebellar GrC layer (Maex and De Schutter, 1998). GENESIS simulation scripts for this model were obtained from http://www.tnb.ua.ac.be/models/network.shtml, and the network specified by these scripts, consisting of 12 mossy fiber (MF) inputs, 75 GrCs, and 4 Golgi cells (GoCs), was recreated in *neuroConstruct* (Figure 6A). The GrC model outlined previously (Figure 4), and a GoC model, also with ChannelML-based channel mechanisms, were used in the network (Experimental Procedures). A key conclusion from the original study was that GrC firing becomes entrained by GoC feedback inhibition during random MF input. Figure 6B shows population spike time histograms of the original model (left) and the model generated in *neuroConstruct*. Although there are minor differences in the exact spike times due to subtle differences in the connectivity of the two models, they both exhibit synchronized GoCs spiking after ∼100 ms, while the GrCs fire in a small time window before these spikes.

Since neurons in the GrC layer model had only a single compartment, we also recreated a 527 cell model of the dentate gyrus (Santhakumar et al., 2005), which had four types of multicompartmental neurons, with 11 channel mechanisms and section specific connectivity, albeit in 1D (Experimental Procedures; Figure 6C). A key result from the model was to show that increased MF connectivity (sprouting) could generate epileptiform network activity. Figure 6D shows the raster plots of spike times for the original model and *neuroConstruct* versions of the model. Focal stimulation at 5 ms caused the central 100 granule cells to fire synchronously a short time later, producing the initial line in the plot. The extra connectivity in the network that mimicked MF sprouting caused the activity to propagate to the other cells in the network over approximately 200 ms in both the original and *neuroConstruct* version. These results show that *neuroConstruct* can faithfully reproduce two of the most advanced published network models, demonstrating its ability to recreate models of different brain regions and the validity of the internal implementation of cell mechanisms and neuronal connectivity.

#### Extension of the 1D Cerebellar Granule Cell Layer Model to 3D

**Figure 7:**
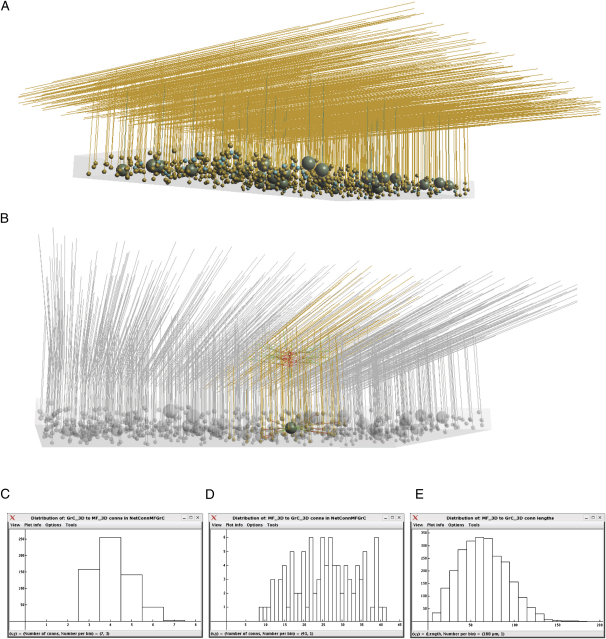
Extension of a 1D Model of Granule Cell Layer to 3D (A) Visualization of a 3D cerebellar GrC model based on a published 1D model (Maex and De Schutter, 1998). MF terminals (blue), GrC somas (orange), and GoC somata (green) are packed in a 3D region (500μm in PF direction, 1 mm parasagittally, 50 μm in thickness) representing a section of the GrC layer of the cerebellar cortex. The ascending segments and parallel fibers of the GrCs extend into the molecular layer region, as do the single dendrites of the GoCs. (B) A single GoC and associated network connections highlighted using the transparency option in *neuroConstruct*. The range of connection lengths is larger than the experimentally reported values for the GoC dendritic tree (∼200 μm [Dieudonne, 1998]) due to the reduced cell density. (C) Histogram of the distribution of number of synaptic connections received by GrCs from MF terminals. Axis variables shown at bottom of window in (C)–(E). (D) Histogram showing the distribution of numbers of synaptic connections made to GrCs by the 96 MFs in the network. (E) Distribution of distances between connected MF terminals and GrC somata, corresponding to dendritic length.

To test the ability of *neuroConstruct* to generate network models in 3D space, we extended the 1D model of the cerebellar GrC layer to 3D. For comparison, we used the same parameters for the cell and synaptic mechanisms as in the original model (Maex and De Schutter, 1998). The model consisted of a 3D region representing the GrC layer (Figure 7A). Thirty-two GoC bodies were packed randomly into this region, and 96 MFs, represented by a single segment for each glomerulus, were placed around these. Finally, 600 GrCs with bifurcated axons were packed randomly, avoiding the space taken up by the existing cell bodies. The axons of the GrCs and the GoCs' single dendrites projected to the molecular layer region. Synaptic connections were generated with the morphology-based connection algorithm (Experimental Procedures). Figure 7B illustrates one of the visualization features of *neuroConstruct*, to highlight the connections of a single cell. We used the anatomical analysis functions in *neuroConstruct* to verify the network connectivity by comparing the properties of the model to measured anatomical properties. These include the number of connections made by the pre- and postsynaptic cells (Figures 7C and 7D) and the distances between the GrC somata and the MF terminals, which correspond to dendritic length (Figure 7E). In this case, dendrites were substantially longer than for real GrCs (Eccles et al., 1967; Ito, 1984) because the GrC density was reduced to below the biologically realistic value for illustrative purposes. This example demonstrates that network models can be created in 3D and that their anatomical properties can be directly compared to measured anatomical parameters.

#### Simulation Management

In addition to specifying the cell and network structure, information on simulation duration, time step, and numerical integration method can be specified through the application interface. Moreover, parameters can be specified for saving and/or plotting during a simulation (e.g., membrane potential, ion channel conductance/current/state variables, calcium concentration, spike times, etc.). It is also possible to specify ranges of parameters (e.g., stimulation amplitude or duration) over which to run multiple simulations, allowing basic parameter space searches. Before a simulation is run, a number of validity checks can be carried out (morphology compliance, appropriate simulation time step/temperature during simulation, etc.) to catch potential errors in neuronal simulations. A *neuroConstruct* project can contain a number of simulation configurations, each of which specifies a subset of cell groups, connections, inputs, etc. to generate, illustrating different aspects of the modeled system. Simulation scripts are automatically generated by mapping the internal representation of the model into the native format of each of the supported simulators. The simulations are initiated through the *neuroConstruct* interface and run on standard versions of NEURON or GENESIS. There is no interaction between the simulator and *neuroConstruct* during the numerical integration.

#### Analysis of Network Activity

**Figure 8:**
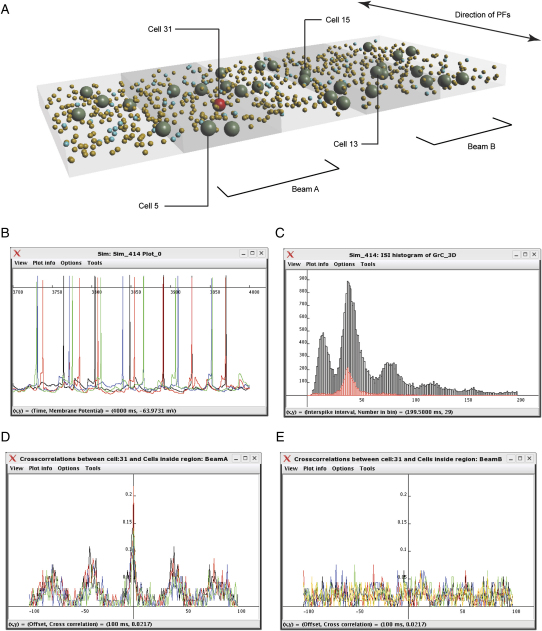
Network Analysis of a 3D Granule Cell Layer Model (A) View of 3D cerebellar GrC layer model showing only the cell bodies. Two regions are identified, beam A and beam B, which have nonoverlapping sets of PFs. (B) Voltage traces of four GoCs at the end of a 4 s simulation run, with network connectivity as outlined previously and 50 Hz Poisson input to the MFs. Black trace (cell 31) and red trace (cell 5) are from GoCs in beam A. Cells 13 (blue) and 15 (green) are in beam B. Axis variables shown at bottom of window in (B)–(E). (C) Interspike interval histograms of the GrCs (black) and GoCs (red). The peak at approximately 40 ms reflects the observed average firing rate of the GoCs of 23.8 Hz, the single peak resulted from regular GoCs spiking. The GrCs have a lower average firing rate and do not fire on every GoC cycle, hence the multiple peaks in the histogram. (D) Crosscorrelation between cell 31 and the other four GoCs in beam A, each color graph representing a different cell. The y axis represents the probability of a spike occurring in the other cell with the specified offset (1 ms time window). (E) Crosscorrelation between cell 31 and the six GoCs in beam B, with identical axes to D.

Saved simulations can be browsed and loaded through an interface in *neuroConstruct*. The simulation can be replayed at various levels of detail, and there are several features for analyzing network behavior. Figure 8A shows the 3D GrC layer model with cell processes and connections removed for clarity. At the most basic level, the voltage of specific cells can be plotted (Figure 8B) or a raster plot of spike times generated (as in Figure 6D). A histogram of interspike intervals shows that GoCs (Figure 8C, red) and GrCs (Figure 8C, black) in the 3D network model fire with similar intervals as for the 1D model (Figure 6B), with the multiple peaks in the GrC histogram reflecting the fact that GrCs do not fire on every GoC cycle. Interestingly, our simple 3D GrC layer model also exhibited spatiotemporal properties that are not present in the 1D model. To quantify these properties, we defined two analysis regions, with cells which shared different PF inputs (Figure 8A); GoCs in these regions (beam A and beam B) connect with largely nonoverlapping sets of GrCs (Figure 7B). As Figure 8B shows, APs from cells in the same beam were more closely aligned at the end of the simulation than in different beams (black and red traces, blue and green traces). We investigated this further by comparing the correlation in spike times over the whole 4 s simulation run between a GoC in beam A (cell 31, Figure 8A, red) with other GoCs in the same beam and GoCs in beam B. A higher correlation was found between this cell and the four other GoC cells in beam A (Figure 8D) than with the six GoCs in beam B (Figure 8E). This behavior is consistent with experimental results comparing simultaneous recordings from GoCs along and across PF tracts (Vos et al., 1999). This simple model demonstrates that network models can be generated and analyzed in *neuroConstruct* with more realistic anatomical properties and behaviors than have been achieved previously.

*neuroConstruct* also automatically creates files for loading data into common numerical analysis packages for more specialized analysis. Script files are included for quick analysis (e.g., for generating raster plots, spike histograms, etc.) in MATLAB or GNU Octave (http://www.octave.org), an open source application compatible with MATLAB script files. Files are also generated for importing simulation data into IGOR Pro. These can be analyzed with NeuroMatic (http://www.neuromatic.thinkrandom.com), an open source set of functions for IGOR Pro, specifically for analysis of electrophysiological data. Experimental data traces can also be imported into *neuroConstruct* for direct comparison with simulation data.

## Discussion

*neuroConstruct* is a new platform-independent software tool for constructing, visualizing, and analyzing conductance-based neural network models with properties that closely match the 3D neuronal morphology and connectivity of different brain regions. A user-friendly GUI allows models to be built, modified, and run without the need for specialist programming knowledge, providing accessibility to both experimentalists and theoreticians studying network function. Models built with *neuroConstruct* are simulator independent and can be automatically mapped onto the NEURON or GENESIS simulation environments for numerical integration. Model components are stored in a simulator-independent XML format, allowing interchange and reuse of model components across simulators. We have demonstrated the functionality of *neuroConstruct* by creating and analyzing a simple 3D network model of the cerebellar GrC layer.

### Construction of 3D Neural Network Models

Quantitative measurements of network properties including cell densities, numbers of synaptic connections between cell groups, and dimensions of axonal and dendritic fields are available for several brain regions including cortex (Douglas and Martin, 2004; Somogyi et al., 1998) and cerebellum (Harvey and Napper, 1991; Sultan and Bower, 1998). However, generating biologically realistic 3D neuronal network models from such data has proven difficult using the direct scripting approach. This is because, unlike many random artificial networks, networks of neurons in the brain exhibit inhomogeneous connectivity probabilities (Lubke et al., 2003), spatial clustering, and an enhanced probability of certain multicell motifs (Song et al., 2005; Sporns and Kotter, 2004). These are due to a prevalence of local connections and the presence of local circuits (Yoshimura and Callaway, 2005), which are thought to be essential for local computations (Pouille and Scanziani, 2004).

Several core functions within *neuroConstruct* facilitate the generation of 3D network models with increased biological realism. These include the ability to import neuronal reconstructions in multiple file formats and the automated placement of cells in defined 3D patterns. Two algorithms enable synaptic connectivity to be generated in 3D space with subcellular specificity. The first was designed for cell models with fully reconstructed axons, axons that are rather invariant (e.g., PF-PC and Schaffer collateral-CA1 synaptic connections [Shepherd, 1998]) and large terminals that innervate many postsynaptic cells (e.g., cerebellar MFs). The second is designed for cells with dense axonal arborizations that project over a particular region of 3D space (e.g., spiny stellate cells in cortex [Lubke et al., 2003] and various interneurons in cortex, hippocampus, and cerebellum [Shepherd, 1998]). Nonuniform network connectivity can be implemented in *neuroConstruct* by defining multiple groups of cells and/or connections and by applying connection probabilities that decay with distance. This potentially allows local circuits with spatially correlated synaptic connectivity, feed-forward inhibitory networks (Yoshimura and Callaway, 2005), and networks with “small world” properties (Watts and Strogatz, 1998) to be created. Also, highly skewed distributions of synaptic weights (Song et al., 2005) could be implemented. This flexibility in the generation of circuits should allow a wide range of spatially nonuniform local circuits to be generated in *neuroConstruct*.

### Model Accessibility and Reuse

The accessibility of large-scale neural network models is currently limited by the fact that they are large specialized programs, often written in different languages, which run on different simulators (Maex and De Schutter, 1998; Santhakumar et al., 2005; Traub et al., 2005). Modifying and rerunning such programs can be difficult and requires specialist programming knowledge. While recent efforts have been made to improve accessibility with the development of GUI interfaces in NEURON and GENESIS, network models are usually written and run using script files. We have addressed this issue by developing a GUI for *neuroConstruct* that allows networks to be built, visualized, and analyzed. Moreover, *neuroConstruct* automatically writes the simulation code and runs it on the chosen simulator (NEURON or GENESIS). No programming knowledge is therefore needed to create, run, and analyze a large network simulation using *neuroConstruct*. These features of *neuroConstruct* make neural network simulations more accessible to nonprogrammers, thereby providing a new tool for both research and teaching.

The latest NeuroML specifications (Crook et al., 2007; Goddard et al., 2001) form the core of our simulator-independent model descriptions. Key advantages of using XML are the facilitated exchange of information between different applications, the simple validation of files, and the ability to include structured metadata describing the contents of the files. Increased adoption of these standards, which are also used in the latest version of NEURON and which will form the basis of model descriptions in GENESIS 3/MOOSE (currently under development at http://sourceforge.net/projects/moose-g3), will promote greater model reuse and collaboration between research groups on cellular and network models (Cannon et al., 2007).

### Current Limitations and Future Developments

*neuroConstruct* presently generates models that can be run on single-processor machines. The scale of simulations that can be run and visualized is therefore limited by the processor and video memory, respectively. We have run simulations of up to 5,000 multicompartmental neurons (50,000 simulated compartments) on a single processor with 2 GB of memory. This simulation could be visualized with a 128 MB video card. For larger simulations, the processor and video memories would have to be scaled up accordingly (we have visualized 50,000 multicompartmental neurons with a 256 MB video card and 8 GB of RAM). Simulations such as that illustrated in Figure 8 (728 compartments) can take 1–2 hr for a 4 s simulation on a single processor, so if larger-scale simulations or extensive parameter searching is required, parallelization may be necessary. We plan to include in the next major release of *neuroConstruct* features for distributing multiple individual simulations using CONDOR (http://www.cs.wisc.edu/condor) and parallelization of large network simulations using the recently developed parallel version of NEURON (Migliore et al., 2006), which is being used for simulations in the Blue Brain Project (Markram, 2006). Parallel simulations will also be a key feature of GENESIS 3/MOOSE, which will be supported in future versions of *neuroConstruct*.

Functions beyond the scope of the *neuroConstruct* GUI interface can be added by inserting native NEURON or GENESIS code. We intend to improve the flexibility of *neuroConstruct* by including a Python-based (http://www.python.org) scripting interface. This will allow greater access to the internal variables of a model, allowing easier parameter searching and model optimization. *neuroConstruct* is closely linked to the NeuroML initiative, and future extensions to ChannelML will allow the implementation of new channel types and plasticity mechanisms and will be more compatible with systems biology standards (Finkelstein et al., 2004) such as with SBML (Hucka et al., 2003) and CellML (Lloyd et al., 2004). This opens the possibility of interaction with 3D diffusion-reaction packages like MCell (http://www.mcell.cnl.salk.edu) and VCell (http://www.nrcam.uchc.edu), although the difference between the morphological representations in these and compartmental neuronal simulators could be restrictive. On the network connectivity side, work is ongoing in the NeuroML project with the developers of Topographica (Bednar et al., 2004), NEST (Diesmann and Gewaltig, 2002), and Neurospaces (Cornelis and De Schutter, 2003), to gain a broad consensus on descriptions of network connectivity.

### Toward More Realistic Models of Brain Function

The modular structure of *neuroConstruct* allows the addition of new features which extend the cellular and network model representations, ensuring compatibility with future advances in our understanding of brain mechanisms. For example, the 3D spatial information can be used to investigate the role of diffusion in brain function. We have implemented a simple model of a diffusible substance that transiently depresses synaptic conductances (Figure S2). Although oversimplified, this proof of concept simulation illustrates how *neuroConstruct* could be used to examine volume-signaling mechanisms such as NO or the relationship between metabolism and neuronal activity, which underlies functional imaging (Attwell and Iadecola, 2002). Extensions currently envisioned that will allow greater biological realism include automated generation of heterogeneous cell morphologies, using approaches similar to L-Neuron (Ascoli et al., 2001) or NeuGen (Eberhard et al., 2006). Moreover, detailed reconstruction of large blocks of tissue using serial block face scanning EM could provide more accurate information about the 3D circuit topology and local spatial arrangements of synapses. Indeed, it even opens the possibility of including ultrastructure at 30 nm resolution (Briggman and Denk, 2006), which would allow more detailed diffusion models. If such EM data stacks were converted into compartmentalized anatomical objects and stored in MorphML format, they could then be directly imported into *neuroConstruct* and used to build network models. This combination of technologies would open the possibility of 3D network modeling with unprecedented levels of biological realism.

### Conclusion

By providing a tool for building, visualizing, and analyzing network models in 3D space using a user-friendly GUI, without the need for programming, *neuroConstruct* increases the accessibility of modeling brain function. The new functionality and accessibility provided by *neuroConstruct* makes it suitable for both experimentalists and theoreticians. It can also be used for teaching network function in health and disease. The 3D models generated will allow simulations of increased biological realism, enabling more direct comparisons with results from new experimental methods for measuring neural activity in 3D at high spatial and temporal resolution.

## Experimental Procedures

### Morphology

*neuroConstruct* uses a simulator-independent representation of neuronal morphology, which allows translation between simulator-specific formats. In NEURON, unbranched neurites are specified in 3D by a sequence of points and diameters, outlining their shapes (termed sections). Membrane surface area and axial resistance are computed from these values. Sections can be subdivided into evenly spaced segments (by specifying the variable *nseg*) for numerical integration, which is carried out at their center points. In contrast, GENESIS uses a single unit, termed a compartment, as the building block for both morphology and numerical integration. *neuroConstruct* defines a section (which maps directly onto a NEURON section) as an unbranched part of the neuronal morphology with uniform biophysical properties. Sections contain one or more segments whose endpoints give the 3D structure along the section. Note that the number of anatomical segments in *neuroConstruct* is not the same as *nseg* in NEURON. Instead, the *nseg*/spatial discretization value is a property of the *neuroConstruct* section. For GENESIS, each *neuroConstruct* segment is mapped to a single compartment.

Manual editing of the imported morphologies is possible, and *neuroConstruct* can also recompartmentalize neurons, allowing morphologies originally in Neurolucida or NEURON format (e.g., 4000–5000 segments) to be mapped onto a reduced number of segments/GENESIS compartments (∼500–1000; Figure S1). Overall cell structure is preserved, and each section (e.g., five to ten segments) is mapped onto two single-segment cylindrical sections (Figure 2B) corresponding to GENESIS compartments. The radii of the cylinders are calculated to preserve cell membrane surface area, total length, and axial resistance along sections. There is a one-to-one mapping between *neuroConstruct* and MorphML format (Crook et al., 2007); segments are mapped to segment elements, and sections are mapped to cable elements. Automatic checks in *neuroConstruct* signal potential problems with morphologies including dendritic segments of zero diameter or zero length and dendrites that are detached from the cell body.

### Cell Mechanisms

Models of cell mechanisms can be specified using a simulator-independent ChannelML description (further examples can be obtained from http://www.morphml.org:8080/NeuroMLValidator) or by a simulator-dependent native script language (NMODL or GENESIS script) which creates and sets the parameters for the object enabling the mechanism. For NEURON, the NMODL files are compiled automatically before the simulation is run.

### Connectivity Algorithms

Connections can be defined relative to the pre- or postsynaptic cell. Each source cell is assigned a number of connections, which can be fixed or variable within set bounds. One or more synaptic mechanisms are associated with the connection, and these can have variable or fixed weights and internal delays. With the morphology-based connection, the target cell can be chosen at random, can be the closest available cell, or the closest cell from a random pool of n possible locations. Maximum and minimum connection lengths can also be set. It is often convenient to calculate the time the AP takes to get from the soma to the synaptic terminal, rather than model the axonal sections explicitly, to reduce computational overhead. AP propagation speed can be specified for cells, and *neuroConstruct* will calculate the extra synaptic delay from the axonal morphology. For the volume-based connection, an axonal arborization volume is defined and any appropriate target segment falling within this region is a candidate for a connection. Nonuniform connectivity is generated by assigning putative connection locations a connection probability that is a function of radial distance or x, y, z coordinates relative to the source soma. The spatial-dependence function can be defined by the user. A connection is made if a random number (0–1) is ≤ the connection probability; otherwise, another random location is picked until all connections are made.

### Network Model Details

In the 1D GrC model, MFs were modeled as single compartments, firing Poisson trains of random spikes. Twelve MFs were placed along a 900 μm line, and the 75 GrCs and 4 GoCs were displaced vertically to facilitate visualization. Synaptic connections between MFs and GrCs had both AMPA and NMDA receptor synaptic mechanisms, with random weights (multiplicative factors of a physiological synaptic conductance) of 5.1–6.9 and 3.4–4.6, respectively. Each GrC was connected to four random MFs within a horizontal distance of 400 μm, giving a radius of influence of five times MF separation. This differed from the combinatorial expansion in the original model but produced an equivalent number of MF-GrC inputs, a similar radius of influence for each MF and more anatomical realism in the stochasticity of the connection. Each GrC connected to all four GoCs via an AMPA receptor synaptic mechanism with a random weight 0.51–0.69. The inhibitory GoCs-GrC connections had a GABA_A_ receptor synaptic connection with a random weight of 38.25–51.75, and each GrC was connected to closest GoC. In our 3D model, the number of cells in the GrC layer reflects a scaling up of the 1D network model by eight times. GrCs consisted of a soma and a bifurcated axon which formed the parallel fibers (PFs). The segments for these axons were not explicitly simulated; an AP propagation speed was specified to provide the extra synaptic delay. GoCs consisted of a soma, taken from the previous model, together with a single dendrite of similar length to the ascending segment of the GrC. The number of MF connections to each GrC was taken from a truncated Gaussian distribution (max 7, min 3, mean 4), reflecting experimentally measured numbers (Eccles et al., 1967).

The dentate gyrus model implemented in *neuroConstruct* (Figure 6C) was based on a topographic strip rather than the ring (see Figure 3 of Santhakumar et al. [2005]) used to eliminate edge effects. Postsynaptic target cells were selected by setting maximum and minimum distances for synaptic connection lengths along the line of cell bodies using the morphology-based connection algorithm. Since only 5 of the 11 channel mechanisms were covered by the ChannelML specifications, we reused existing NMODL files for the remaining channels. The original model was downloaded from http://senselab.med.yale.edu/senselab/modeldb/ShowModel.asp?model=51781, and the perforant path inputs were positioned at GrCs 200–299, to facilitate comparison to the *neuroConstruct* model. Network generation in *neuroConstruct* resulted in similar means but some differences in the standard deviations of synaptic convergence (Table 3 of Santhakumar et al. [2005]). The small divergence of behavior when the wave of activation reached the end of the strip (Figure 6D) is also due to the change from a ring structure to a linear network topology.

## Supplemental Data

Document S1. Two Figures
