# Supplementary material for: neuroConstruct: A Tool for Modeling Networks of Neurons in 3D Space
Source: Neuron. 2007 Apr 19;54(2):219–35. doi: 10.1016/j.neuron.2007.03.025 (PMC1885959; doi:10.1016/j.neuron.2007.03.025)
Supplement: Document S1. Two Figures [file mmc1.pdf]

Neuron, Volume *54*

Supplemental Data

neuroConstruct: A Tool for Modeling

Networks of Neurons in 3D Space

Padraig Gleeson, Volker Steuber, and R. Angus Silver

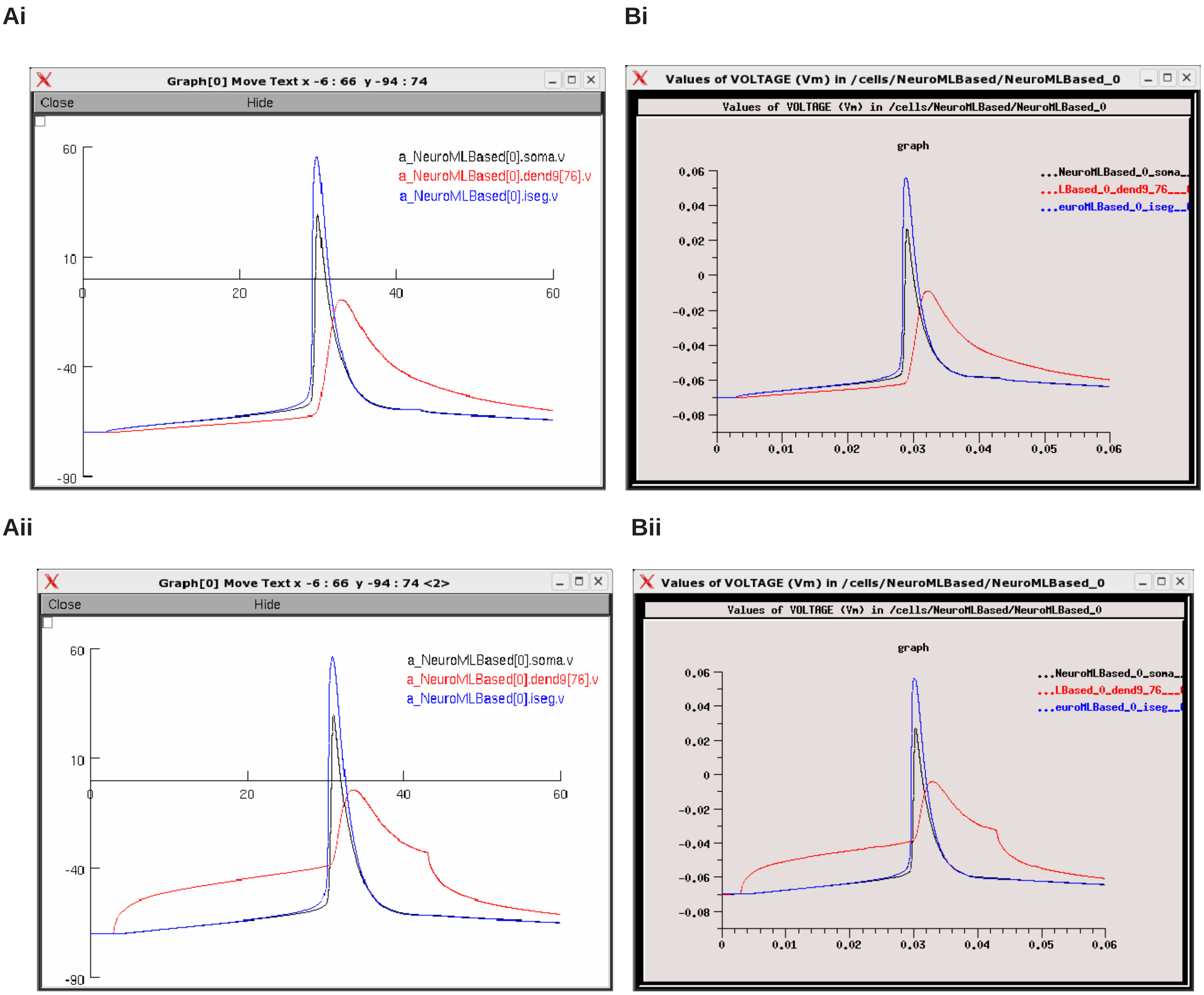

Figure S1. Comparison of the Properties of a Detailed Model of a Layer 5 Pyramidal Cell Implemented in NeuroML and Run on the NEURON and GENESIS Simulators

A model of a rat layer 5 pyramidal cell from Mainen et al. (1995; illustrated Figure 2A) was converted from the original NEURON script (obtained from http://senselab.med.yale.edu/senselab/modeldb/ShowModel.asp?model=8210) to *neuroConstruct's* NeuroML-based internal morphological representation with channels that were specified in ChannelML. Minor changes were made to fix zero length sections, etc. This allowed the model to be run on both NEURON and GENESIS simulators.

(A) Voltage traces from the layer 5 pyramidal cell model implemented in *neuroConstruct* and run on the NEURON simulator. (Ai) The cell response to a somatic current step at the soma (black trace), initial segment (blue) and a point along the main apical dendrite at 416 µm from the soma (red). Axes are voltage (mV) and time (ms). (Aii) shows the responses, at the same locations, to stimulation at the dendritic location. These results reproduced those in Figure 3A in Mainen et al. (1995).

(B) The same layer 5 pyramidal cell model implemented in *neuroConstruct* and run on the GENESIS simulator. The colored traces correspond to those described in (A). Apart from a minor difference in the timing of the somatic spike (<1 ms) the properties of the model closely reproduce those obtained in with NEURON. Axes are voltage (V) and time (s).

We also tested a version of this model when the original morphology of 5726 segments was recompartmentalized in *neuroConstruct* to 800 segments, while maintaining the overall segment length, total surface area and total axial resistance (Experimental Procedures). Simulation in GENESIS using the recompartmentalized morphology produced similar results to those obtained in B (with a small temporal shift ~1 ms) and speeded up the simulation by a factor of 10.

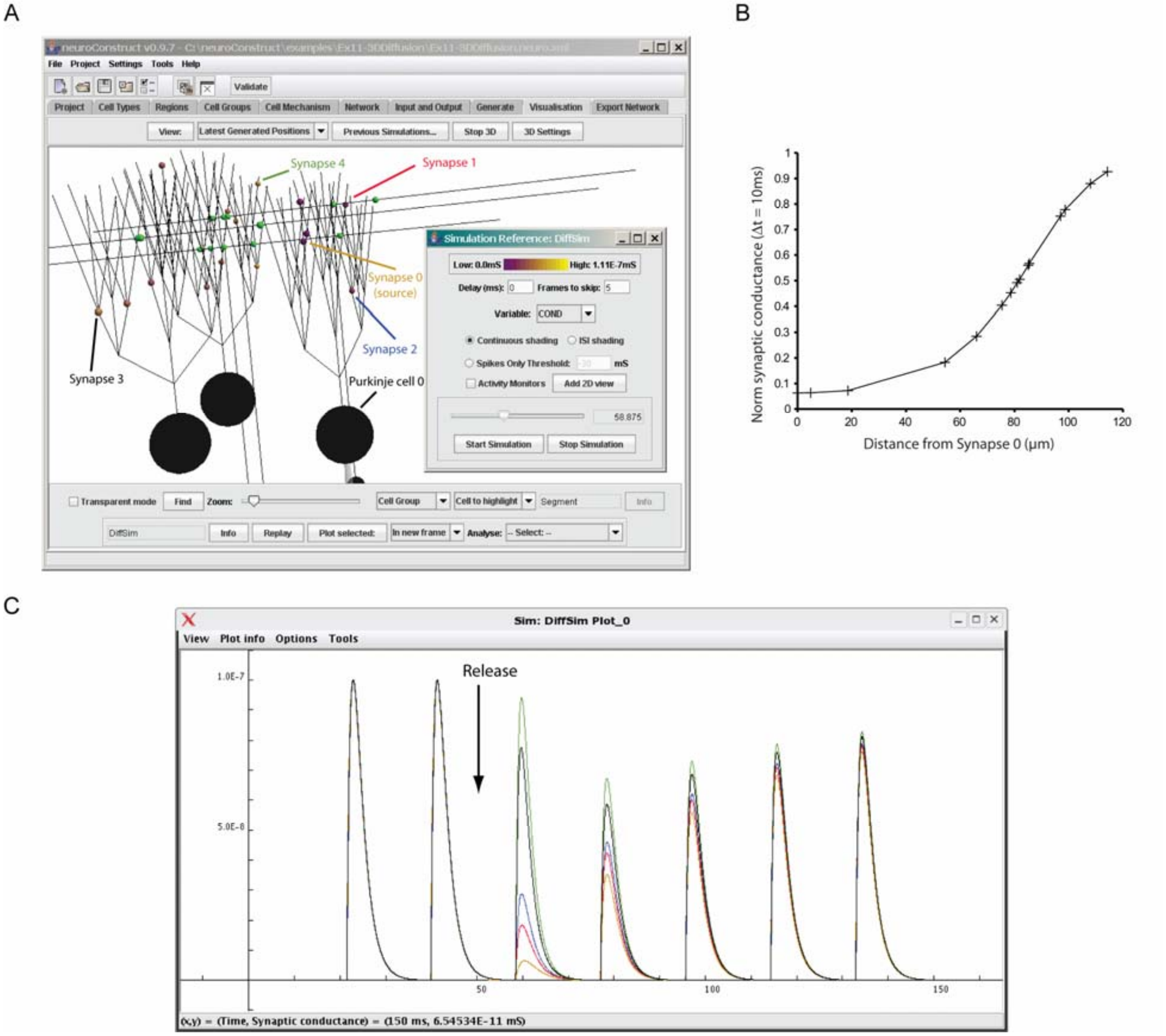

Figure S2. Example of a Simple Diffusion Mechanism Implemented in a 3D Network Model

To illustrate how 3D information in *neuroConstruct* network models can be used we have created a simple 3D diffusion model of substance that transiently inhibits synapses on Purkinje cells (PCs). This was implemented by writing a new synaptic mechanism in NEURON NMODL script and inserting it into the *neuroConstruct* model via the GUI interface. In the simulation shown above a diffusible substance was released from an individual synapse. All other synapses registered this event (via the setpointer call in NEURON). The concentration of the diffusible substance was calculated at each synaptic location with an analytical solution for a point source in an infinite medium (Equation 3.5; Crank (1975) The Mathematics of Diffusion). The inhibitory effect of the diffusing substance on the synaptic conductance was implemented with a normalized scale factor that was calculated using a Hill expression for a first order reaction.

(A) A *neuroConstruct* screenshot of three parallel fibres (PFs; granule cell axons) and three Purkinje cells (PCs) arranged in 3D. Green spheres show presynaptic locations and yellow to purple colour coding shows the conductance values of the postsynaptic locations as indicated in the simulation replay panel. For illustrative purposes we allowed PFs to make multiple synaptic contacts on an individual PC in this model.

(B) A plot of the peak synaptic conductance for all 15 synapses at 10 ms after release (60 ms on panel C), normalized to pre release peak conductance. At this early time the inhibitory substance had not diffused very far and distant synapses are only slightly affected.

(C) A plot of PF-PC synaptic conductances as a function of time for the five highlighted synapses shown in A during regular stimulation of the granule cells. At 50 ms, a pulse input was applied to Purkinje Cell 0 causing it to fire. This triggered the release of an inhibitory substance from Synapse 0, which had been active within a short time interval. By the subsequent

synaptic activation (10 ms later) the synaptic conductances were inhibited with the greatest effect at the closest synapses. At later times the conductance amplitudes converged and then recovered back to control.

This simple, albeit rather unphysiological model, demonstrates that diffusion mechanisms can be implemented in 3D network models created by *neuroConstruct*, but at present this requires the insertion of custom NEURON code.
